# Revisiting the Non-Coding Nature of Pospiviroids

**DOI:** 10.3390/cells11020265

**Published:** 2022-01-13

**Authors:** Konstantina Katsarou, Charith Raj Adkar-Purushothama, Emilios Tassios, Martina Samiotaki, Christos Andronis, Purificación Lisón, Christoforos Nikolaou, Jean-Pierre Perreault, Kriton Kalantidis

**Affiliations:** 1 Department of Biology, University of Crete, Vassilika Vouton, 71409 Heraklion, Greece; emiliostassios@gmail.com (E.T.); christoforos.nikolaou@gmail.com (C.N.); 2 Institute of Molecular Biology and Biotechnology, Foundation for Research and Technology, 71110 Heraklion, Greece; christos_andronis@imbb.forth.gr; 3 RNA Group/Groupe ARN, Département de Biochimie, Faculté de Médecine des Sciences de la Santé, Pavillon de Recherche Appliquée au Cancer, Université de Sherbrooke, Sherbrooke, QC J1E 4K8, Canada; charith.adkar@usherbrooke.ca (C.R.A.-P.); jean-pierre.perreault@usherbrooke.ca (J.-P.P.); 4 Biomedical Sciences Research Center “Alexander Fleming”, Institute for Bioinnovation, 16672 Vari, Greece; samiotaki@fleming.gr; 5 Instituto de Biología Molecular y Celular de Plantas (IBMCP), Consejo Superior de Investigaciones Científicas (CSIC), Universitat Politècnica de València (UPV), Ciudad Politécnica de la Innovación (CPI) 8 E, Ingeniero Fausto Elio s/n, 46011 Valencia, Spain; plison@ibmcp.upv.es

**Keywords:** viroid, translation, non-coding RNA, cirRNA, mass spectrometry, PSTVd

## Abstract

Viroids are small, circular, highly structured pathogens that infect a broad range of plants, causing economic losses. Since their discovery in the 1970s, they have been considered as non-coding pathogens. In the last few years, the discovery of other RNA entities, similar in terms of size and structure, that were shown to be translated (e.g., cirRNAs, precursors of miRNA, RNA satellites) as well as studies showing that some viroids are located in ribosomes, have reignited the idea that viroids may be translated. In this study, we used advanced bioinformatic analysis, in vitro experiments and LC-MS/MS to search for small viroid peptides of the PSTVd. Our results suggest that in our experimental conditions, even though the circular form of PSTVd is found in ribosomes, no produced peptides were identified. This indicates that the presence of PSTVd in ribosomes is most probably not related to peptide production but rather to another unknown function that requires further study.

## 1. Introduction

The ‘central dogma’ of molecular biology explains the flow of genetic information and consists of the process of transcribing DNA into RNA, which is then translated into proteins. Translation is usually divided into four stages: initiation, elongation, termination and ribosome recycling [1]. The initiation step is the most complex in terms of the proteins involved. During initiation, the 40S ribosomal subunit binds to the mRNA and scans until an initiation codon (AUG) is found. In the last few years, several alternative initiation starting codons have been described [2]. Following initiation, the 60S ribosomal subunit joins to form the 80S ribosome whereupon the elongation step starts, translating the information encoded in three consecutive nucleotides into an amino acid (aa), creating a peptide, and then a protein. Recognition of the stop codon drives the termination process and the release of the protein. Finally, ribosome recycling occurs, where the messenger RNA (mRNA) is released and the 80S ribosome is separated into its 40S and 60S components [1].

For many years, it was believed that mRNAs were the only RNAs produced by DNA that can be translated. However, only around 4% of the RNA transcribed is actual mRNA [3]. The remainder corresponds to different classes of non-coding RNAs [4]. In 1979, a peculiar endogenous circular RNA (circRNA) was discovered in HeLa cells [5]. At the time, this was considered an exception, but nowadays, especially with the technological breakthrough of high-throughput sequencing (HTS) technologies, circRNAs are considered as a large class of ubiquitously expressed RNA molecules. circRNAs are produced through splicing and regulated by specific mechanisms [6,7]. Their best characterized role is to act as miRNA and protein sponges, thus altering gene expression [7].

In the last few years, the idea that circRNAs can be used as templates for protein synthesis has emerged [8,9,10,11]. Some circRNAs include in their sequence an internal ribosome entry site (IRES), which constitutes a highly structured domain containing multiple stem loops, to allow initiation of translation [9,10]. Moreover, it has been proposed that other regions of circRNAs, named IRES-like domains, can also be used for translation initiation [12]. The translation of circRNAs produces small peptides of fewer than 100 aa, termed microproteins or non-conventional peptides (NCPs), discovered mostly with the use of mass spectrometry [12]. In humans, these microproteins seem to be extremely abundant in the heart, liver and kidney, as suggested by translatome analysis [13].

The first circRNA of exogenous origin discovered was a viroid, whose circularity was confirmed by electron microscopy in 1973 [14]. Viroids are plant pathogenic single-stranded, circular, non-coding RNA molecules capable of infecting a diverse range of host plants of economic importance [4,15]. With their size ranging between 246–401 nucleotides (nt) and no capsid, they are considered as one of the smallest and simplest pathogens of life. They were first discovered in 1971 in potato (i.e., potato spindle tuber viroid—PSTVd), but since then, more than thirty different viroids have been identified [16,17]. They are divided into two families depending on their structure and their site of replication in host plants [4,18]. *Pospiviroidae* have a rod-shaped RNA genome and replicate in the nucleus via an asymmetric rolling-circle model, whereas *Avsunviroidae* possess a highly branched structure and replicate in chloroplasts via a symmetric rolling-circle mechanism [4,18,19]. In order to establish an infection, viroids need to use all the structural information found in their genome, which include stem loops for interactions with host proteins as well as viroid-derived small interfering RNAs (vd-siRNAs) produced by Dicer-like proteins, even though the mechanism by which this occurs remains poorly understood [20].

Although viroids have long been considered non-coding circular RNAs, in light of the discovery that some circRNAs and other small highly structured RNAs can be translated, the idea that viroids may also be translated reemerged. As an example, a plant circRNA satellite of 220nt, sharing important features with viroids, has been found capable of producing a small peptide of 16KDa [21]. The first studies attempting to answer this question were conducted in 1974, when PSTVd and Citrus exocortis viroid (CEVd) were tested for their ability to be translated using an in vitro translation system, but without success [22,23]. Attempts were also made in vivo with PSTVd-infected tomatoes, CEVd-infected *Gynura aurantiaca*, and CEVd-transfected *Xenopus laevis*, and again, microproteins were not identified [24,25,26]. These works helped establish the belief that viroid RNAs are most probably not translated. On the other hand, in 2019, Cottilli et al., using mainly CEVd-infected tomatoes, showed that viroid RNAs are found in ribosomal fractions, suggesting that at least in terms of localization, viroids are found very close to the translational machinery [27]. Furthermore, direct interaction of eIF1A, an important protein of the translation mechanism, and both CEVd and peach latent mosaic viroid (PLMVd) has been proposed [28,29]. A recent work by Marquez-Molins et al. has reignited the possibility that viroid RNAs might be translated [30].

In the present study, we revisit the question on the translation of viroid RNAs using more advanced techniques than those previously employed. Firstly, we have applied bioinformatics analysis using all available *Pospiviroidae* sequences and in order to identify putative ORFs. We then showed that a portion of PSTVd may localized to ribosomes, mostly in its circular form. Finally, we performed in vitro and in vivo experiments to identify possible viroid-encoded polypeptides. Taken together, by using distinct and more sensitive techniques, we have confirmed the results of classic studies, which indicate that viroids cannot produce any peptides, thus suggesting that viroid localization in proximity of ribosomes is due to reasons other than translation.

## 2. Materials and Methods

### 2.1. Bioinformatic Analysis

Nucleotide sequences of all available strains for 30 viroid species from the *Pospiviroidae* family were downloaded from the NCBI database in FASTA format. Sequences identified as duplicates were excluded from the analysis (Table S1). All the sequences were then analyzed for the existence of potential ORFs according to the following steps:

Open Reading Frame (ORF) detection: ORFs in circular genomes may originate at any point in the sequence and run the length of the genome or even exceed it. To identify candidate ORFs in the circular viroid genomes, we used artificial genome sequences as contigs composed of two copies of the same sequence joined together. All AUG and non-AUG starting codons (according to [2]) were identified in all three reading frames, and sequence strings that started with the detected starting codons and stopped at the end of the remaining sequence were obtained as ORF-containing candidates (putative ORFs). Each such putative ORF was then trimmed to contain contiguous subsequences between in-frame start and stop codons, which were retained for further analysis. In the case of multiple in-frame overlapping ORFs terminating at the same stop codon, only the longest ORF was kept in the final list of candidates.

Translation of ORFs: Each sequence from the final set of putative ORFs was in silico translated into a protein, based on the genetic code. For each viroid species, basic analyses were carried out, including the number of different peptides per species, mean peptide length, standard deviation of peptide length, mean molecular weight of peptides and standard deviation of peptide molecular weight (Table 1). BLASTp analysis was carried out to search for significant sequence similarity (*p* value < 0.05) with previously characterized proteins.

ORF emergence tendencies: To investigate if viroid genomes show a greater ORF frequency than expected by chance, the same procedure was subsequently used on randomly scrambled genome sequences with an identical nucleotide composition. Except from the actual number of ORFs per genome, the localization of the ORFs across the 5 characteristic genome domains, (the terminal left domain, the pathogenicity domain, the central domain, the variable domain and the terminal right domain) was also checked for enrichment in comparison with the scrambled genomes. To acquire the information about the characteristic domains, BED files with the coordinates of the start of each ORF and the coordinates of the domains, whenever available, were created. The intersect tool from the bedtools suite [31] was used to find the overlaps.

Conservation of ORFs: The conservation rate of the ORFs identified in *Pospiviroidae* genomes, inter- and intra-specifically, was obtained with the use of the MAFFT alignment algorithm (Multiple Alignment using Fast Fourier Transform) [32]. The percentage of occurrence of a nucleotide at each alignment position was calculated, and extended conserved regions were defined as areas having at least 40% similarity among the genomes included in the alignment. The occurrence rate of ORFs among the different strains per genome was also calculated by counting the number of strains where a specific ORF is predicted by dividing over the total number of strains of the species.

Detection of KOZAK motif: We generated a positional-specific scoring matrix (PSSM) through a comparison of the matrices for the KOZAK motif, based on [33], and a background matrix, created from 5074 random sequences (a number equal to the viroid sequences used in the study). We then conducted a motif search using a custom R script that scans the target sequence with the PSSM matrix and returns instances of matrix similarity according to an arbitrary threshold of 0.65.

### 2.2. Plants and Infections

Tomato (*Solanum lycopersicum* cv Rutgers; Livingston Seed Co, Columbus, OH, USA) and *Nicotiana benthamiana* plants were infected with either PSTVd^RG1^ (GenBank Acc. No. U23058) or PSTVd^NB^ (GenBank Acc. No. AJ634596.1). Infections were either performed mechanically or via agro-infiltration. For mechanical infections, the dimeric construct of PSTVd^RG1^ was used to synthesize infectious dimeric transcripts as described previously [34]. PSTVd RNA transcript (1 µg) was inoculated into both plant types. All plants were grown in a growth chamber at a temperature of 25 °C with 16 h of light and 8 h of darkness [35]. For agroinfiltration experiments, *N. benthamiana* plants were agroinfiltrated with an *A*. *tumefaciens* GV3101 strain carrying an infectious PSTVd^NB^ dimer, kindly provided by Dr. De Alba and Dr. Flores (Institute for Cellular and Molecular Plant Biology—IBMCP), as described previously [36]. Plants were grown in a glasshouse under ambient temperature and light conditions.

### 2.3. Total Ribosome Isolation, Polysome Fractionation and RNA Preparation

Total ribosomes and polysomes were prepared as previously described [37] with modifications. Actively growing leaf samples (25 g) were frozen in liquid nitrogen and macerated to a fine powder. Two volumes of cold plant extraction buffer (50 mM Tris-HCl (pH 9.0) (Sigma, Burlington, VT, USA), 30 mM MgCl_2_ (Fischer chemicals, Chicago, IL, USA), 400 mM KCl (Fischer chemicals, Chicago, IL, USA), 17% (*w*/*w*) sucrose (Fischer chemicals, Chicago, IL, USA) were added and clarified by passage through DEPC-treated cheesecloth. The resulting extracts were centrifuged at 3000 rpm for 7 min at 4 °C. One-tenth volume of 20% Triton X-100 was added and samples were centrifuged at 12,000 rpm for 20 min. Clear supernatants were then layered (1:1) on a 60% sucrose cushion (20 mM Tris-HCl (pH 7.6), 5 mM MgCl_2_, 510 mM NH_4_Cl, 60% (*w*/*w*) sucrose) and centrifuged at 28,000 rpm for 19 h in a SW28 rotor in a Beckman Coulter ultracentrifuge (Beckman Coulter, Indianapolis, IN, USA). The resulting pellets were carefully rinsed with resuspension buffer (50 mM KCl, 20 mM Tris-HCl (pH 7.6), 5 mM MgCl_2_) and resuspended in 200 μL of the same buffer. The resuspended total ribosomes were fractionated on a 5–50% sucrose gradient by centrifugation at 16,000 rpm for 13 h in a SW28 rotor. The 40S, 60S and 80S ribosomes and the polyribosomes were purified, and the RNAs were extracted as described previously [38]. Briefly, the RNA was precipitated with 5.5 M guanidine HCl (Sigma, Burlington, VT, USA) and ethanol (Commercial alcohols, Toronto, ON, Canada), followed by acidic phenol:chloroform extraction and re-extraction of the supernatant with an equal volume of chloroform. Purified RNAs were treated with DNase I according to manufacturer’s instructions (Promega, Madison, WI, USA). RNA integrity was evaluated using a Agilent 2100 Bioanalyzer (Agilent Technologies, Santa Clara, CA, USA)).

### 2.4. High Throughput Sequencing for Detection of Quasi-Species

The results of small viroid RNA experiments have been described elsewhere [39]. PSTVd-sRNA sequences of PSTVd^RG1^-infected tomato plants (GEO Acc. No. GSM1717894) were analyzed for the presence of potential start codons. Initially, 21-nt long sRNA with a match score of 1 and mismatch cost of 2 to PSTVd^RG1^ were segregated using CLC Genomic Workbench version 4.6 software (https://www.qiagenbioinformatics.com/products/clc-genomics-workbench/version-11-available/ accessed on 8 December 2021) and were then manually re-examined for the presence of AUG codons.

HTS analysis for PSTVd genomes was performed as follows: PSTVd^NB^ agroinfiltrated plants were collected at 3 weeks post infection (wpi) and RNA was extracted as described previously [40]. Following DNAse I (Roche Diagnostics, Basel, Switzerland) treatment and extraction with phenol/chloroform, the integrity of RNAs was assessed using the Agilent 2100 Bioanalyzer. Library construction used the Ion Total RNA-seq Kit (Life technologies-Merk group, Darmstadt, Germany), and sequencing was performed using the Ion Torrent Proton platform. The quality of the raw reads (a total of 21 928 628) before and after the various cleaning steps was assessed with FastQC [36]. Quality and adapter trimming was performed with fastp [41] using the following settings: -q 20 --length_required 21 --cut_tail --cut_front --cut_mean_quality 20. Cleaned fastq files were aligned to the PSTVd^NB^ genome (AJ634596.1) using BBMap [42] with default settings. Aligned reads (54 426) were extracted with samtools view [43]. Nucleotide variants from bam files were produced with quasitools [44], ran as quasitools call ntvar. The resulting VCF file was then used to extract alternative start codons.

### 2.5. cDNA Synthesis, RT-PCR, RT-qPCR and Northern Blot for PSTVd Detection

Following RNA extraction, cDNA synthesis was performed using 250 ng of RNA and SuperScript III reverse transcriptase (Invitrogen, Carlsbad, CA, USA). PCR was carried out using Q5 DNA polymerase according to the manufacturer’s instructions (New England Biolabs, Ipswich, MA, USA). Primers were either designed for this study or published before (Table S2) [45,46]. PCR-produced fragments were cleaned and cloned in pGEM-T vector (Promega, Madison, WI, USA) using the manufacturer’s instructions, followed by sequencing. The resulting sequences were assembled and aligned using the CLC Free Workbench (https://digitalinsights.qiagen.com/products-overview/discovery-insights-portfolio/analysis-and-visualization/qiagen-clc-main-workbench/ accessed on 8 December 2021) and were then manually analyzed.

For the evaluation of the PSTVd titer in both the total RNA extract and the polysome fraction, cDNA was prepared by reverse transcribing 500 ng RNA (SuperScript III reverse transcriptase—Invitrogen, Carlsbad, CA, USA) in the presence of random primers. Three housekeeping genes, specifically the 5.8S, 18S, and 25S rRNAs, were used for normalization, and three biological and three technical replicates were used. The qBASE framework was used for the analysis [47].

The detection of PSTVd by northern blotting was carried out as described previously [34,36].

### 2.6. In Vitro Translation and Immunoblot Assays

In order to perform in vitro translation, both the Wheat Germ Extract kit (Promega, Madison, WI, USA) and the FluoroTect™ GreenLys Labeling System (Promega, Madison, WI, USA) were used according to manufacturer’s instructions with the following modifications. Briefly, the reaction was performed in 25 µL containing 5 µg viroid RNA (specifically, (+) dimeric, (−) dimeric, (+) monomeric and (−) monomeric) and 2 µL of FluoroTect™. The reactions were carried out at 25 °C for 60 min, followed by an incubation at 30 °C for 60 min. The reactions were then terminated by the addition of RNase A (Promega, Madison, WI, USA). For PSTVd-derived translational analysis, 5 µL of the in vitro translation reactions were separated on a 12% SDS-PAGE gel and were then transferred to polyvinylidene difluoride membranes (Bio-Rad Laboratories, CA, USA). Anti- BODIPY™ FL rabbit IgG (ThermoFischer Scientific Inc, Waltham, MA, USA) at a dilution of 1:500 dilution was used to detect the translation according to the manufacturer’s instructions (Invitrogen, Carlsbad, CA, USA), followed by a subsequent incubation with a 1:10,000 dilution of the IRDye 800CW donkey anti-rabbit-IgG polyclonal antibody (LI-COR). The proteins were subsequently visualized using an LiCOR scanner (LI-COR, Lincoln, NE, USA) at 700 nm.

### 2.7. Proteomic Analysis

Sp3-mediated protein digestion: *N. benthamiana* plants were agroinfected with PSTVd^NB^ and upper leaves were collected 4 wpi. Leaf tissue was pooled and homogenized in 4% SDS, 0.1 M DTT, 0.1 M Tris pH 8 lysis buffer. Three biological replicas of each group (either non-inoculated or 4 wpi) were processed using the sensitive sp3 protocol [48]. Additionally, the sp3 protocol was used in parallel for the digestion of the 3 kDa ultra-filtrate (Sartorius AG, Göttingen, Germany) of the leaf extracts in order to assess the lower molecular weight protein portion. The cysteine residues were reduced in 100 mM DTT and alkylated in 100 mM iodoacetamide (Acros Organics, Thermo Fisher Scientific Inc., Waltham, MA, USA). Twenty micrograms of beads (1:1 mixture of hydrophilic and hydrophobic SeraMag carboxylate-modified beads (Cytiva, Marlborough, MA, USA, former GE Life Sciences) were added to each sample in 50% ethanol. Protein clean-up was performed on a magnetic rack. The beads were washed twice with 80% ethanol and once with 100% acetonitrile (Fisher Chemical, Thermo Fisher Scientific Inc., Waltham, MA, USA). The captured proteins were digested overnight at 37 °C under vigorous shaking (Thermomixer, Thermo Fisher Scientific Inc., Waltham, MA, USA) with 0.5 μg Trypsin/LysC (mixture MS grade, Promega, Madison, WI, USA) prepared in 25 mM ammonium bicarbonate. The next day, the supernatants were collected, dried using a vacuum centrifuge (Savant, Thermo Fisher Scientific Inc., Waltham, MA, USA), solubilized in a mobile phase A, sonicated and the peptide concentration was determined through measurement of the absorbance at 280 nm.

In-gel digestion: One hundred micrograms of homogenized leaf tissue was mixed with 500 μL of lysis buffer (100 mM Tris-HCl (pH8), 200 mM NaCl_2_, 1 mM EDTA, 3 mM MgCl_2_, 10% Glycerol, 1 mM DTT, 1 μg/μL PMSF, 10 μL/mL protease inhibitors cocktail set VI (Calbiochem-Merk group, Darmstadt, Germany), 1 μL/mL Tween 20 (Sigma-Merk group, Darmstadt, Germany), and separated on a 15% SDS PAGE. Gel was stained with ‘blue silver’ Coomassie colloidal blue stain (Thermo Fisher Scientific Inc, Waltham, USA) [49], and the lower part of the gel were excised and subjected to classic tryptic-mediated in-gel digestion [50]. Briefly, gel pieces were excised, transferred into small tubes, destained, dehydrated with acetonitrile and then rehydrated with 25 mM ammonium bicarbonate buffer. After repeating the dehydration, rehydration and dehydration cycles, the dried gel pieces were rehydrated with 12.5 ng/µL trypsin in 25 mM (NH_4_)HCO_3_ solution and incubated overnight at 37 °C. Peptides were extracted from each gel piece with 100 μL extraction buffer (1:2 (*v*/*v*) 5% formic acid/acetonitrile) for 30 min at 37 °C, and the solution was dried in a vacuum centrifuge. Finally, the samples were reconstituted in 2% (*v*/*v*) acetonitrile/0.1% (*v*/*v*) formic acid and sonicated in a water bath for 5 min.

LC-MS/MS: Nano-liquid chromatography of the resulting tryptic peptide mixture was carried out using a Ultimate3000 RSLC system configured with an Acclaim pepmap C18 trap column (Thermo Fisher Scientific Inc., Waltham, MA, USA), and a 25 cm-long pepsep nano column (pepsep.com, Marslev, Denmark) for a total of 500 ng of peptides was loaded on the precolumn at a flow rate of 6 µL/min for 4 min with 0.1% formic acid in water. The peptide separation was achieved using 0.1% (*v*/*v*) formic acid in water (mobile phase A) and 0.1% (*v*/*v*) formic acid in acetonitrile (mobile phase B). The flow rate was set to 350 nL/min in the first 12 min of the gradient and 250 nL/min in the main gradient. The gradient was linear from 8% to 28% phase B in 35 min, 28% to 36% in 5 min, 36 to 95% in 0.5 min, staying isocatic for 5 min and then equilibrating at 8% for 10 min at 350 nL/min.

The data acquisition was performed in positive mode using a Q Exactive HF-X Orbitrap mass spectrometer (Thermo Fisher Scientific Inc., Waltham, MA, USA). MS data were acquired in a data-dependent strategy, selecting up to the top 12 precursors based on precursor abundance in the survey scan (*m*/*z* 350–1500). The resolution of the survey scan was 120,000 (at *m*/*z* 200) with a target value of 3 × 10E6 ions and a maximum injection time of 100 ms. HCD MS/MS spectra were acquired with a target value of 1 × 10^5^ and resolution of 15,000 (at *m*/*z* 200) using an NCE of 28. The maximum injection time for MS/MS was 22 ms. Dynamic exclusion was enabled for 20 s after one MS/MS spectra acquisition. The isolation window for MS/MS fragmentation was set to 1.2 *m*/*z*. Three technical replicas were acquired.

Data Analysis: The generated raw files were searched using the MaxQuant Software (1.6.14.0) (MaxPlanck, Germany) [51] using Andromeda, against the predicted proteome based on the *N. benthamiana* Genome v1.0.1 (Niben v1.0.1, containing 56701 proteins, 2015), with the predicted PSTVd ORFs and the MaxQuant common contaminant database. To be accepted for the identification, an error of less than 20 ppm (first recalibration search) and 4.5 ppm tolerance in the main search of peptide mass tolerance was accepted. Up to 2 missed cleavages were allowed and the modifications taken into account were: oxidation (M); acetylation (protein N-term); deamidation (NQ) as variable and carbamidomethylation (Cys) as fixed modifications. Matching between runs and second peptide options were activated. Protein, peptide and “site” identifications were validated at an FDR of 1% using a reversed database. The above data analysis was repeated using an “unspecific” search mode against the predicted PSTVd ORFs, removing the constraint for tryptic generated peptides.

Data visualization: The MaxQuant search engine quantitative (LFQ) results were analyzed and visualized using the Perseus computational framework (version 1.6.10.43) (MaxPlanck, Germany) [52]. The LFQ values were log2 transformed and the proteins were filtered for potential contaminants, reversed hit and those were only identified by site. The biological and technical replicates were grouped into non-inoculated or PSTVd-infected plants and the two groups were filtered based on at least 70% valid values present in at least one group. Remaining empty values were imputed based on normal distribution. The groups were compared using a student t-test using permutation-based FDR calculation (s0: 0.1, FDR < 0.05). The results after statistical analysis were visualized in a volcano graph based on the difference between the two samples expressed in log2(x) versus their statistical significance expressed in −Log10 (*p* value).

Enrichment analysis: Enrichment analysis was carried out on the PlantRegMap web service (http://plantregmap.gao-lab.org/ accessed on 8 December 2021), using the Niben1.0.1 annotations of *N. benthamiana* with threshold *p*-value ≤ 0.01 [53].

## 3. Results

### 3.1. Analysis of the Presence of ORFs in Viroid Sequences

To identify possible ORFs in viroid sequences, we used the nucleotide sequence of 30 different viroid species from the *Pospiviroidae* family, including all available isolates at the time of this study (Table S1). Firstly, we duplicated the sequence of each viroid to avoid ‘premature’ termination of a predicted ORF at the 3`/5` junction of the genomic sequences, since viroids are circular. Secondly, we used both AUG as well as non-AUG start codons, based on the work of Kearse and Wilusz [2]. Finally, in the case of overlapping ORFs, we decided to keep only the longer ORF. With these rules, we showed that all viroids are predicted to produce small peptides with a mean size of peptides for each species ranging from 3 to 15 kDa (Table 1). It is important to note that differences in the observed number of small peptides for each viroid species can be primarily attributed to the different number of isolates available for the analysis (Supplementary Table S1). All predicted peptides were then analyzed using BLASTp against the complete non-redundant NCBI protein database (nr) to test for similarity with known proteins, but none were identified.

Since the presence of an optimal Kozak sequence can enhance the production of a peptide [54], we studied if the predicted ORFs contain an optimum Kozak sequence associated with the identified start codons. For this purpose, we used the motif described in Joshi et al. [33]. As shown in Table 2, 17 PSTVd isolates present a Kozak frame, whereas CEVd, CSVd and CLVd present the same motif but only in a very small number of the tested isolates. This suggests that even though starting codons are present in viroids, only a few of them are highly favorable to be used for translation.

**Figure 1:**
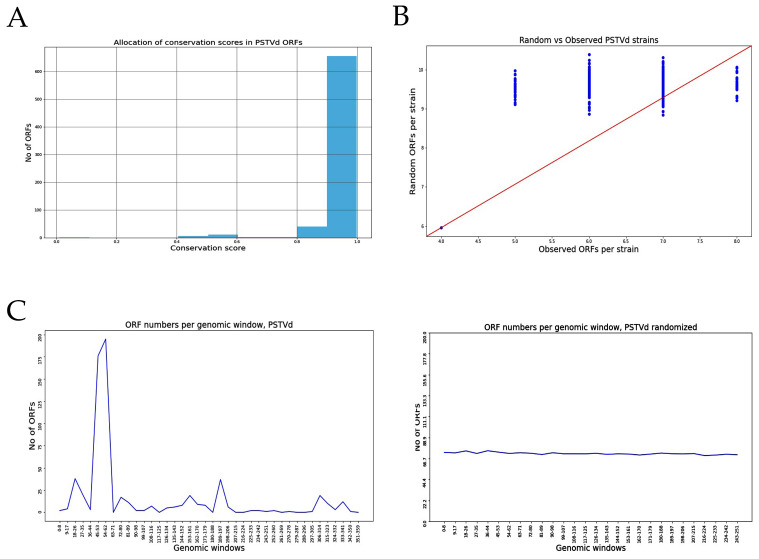
Identification of Possible ORFs in PSTVd. (**A**) Conservation rate in PSTVd isolates. (**B**) Comparison between artificially shuffled genome and real genome for PSTVd. (**C**) Presence of ‘hotspots’ in PSTVd genome.

We then assessed the likelihood of the existence of ORFs in relation to their position throughout the genome. For this, we used two different approaches. Firstly, we calculated the degree of conservation of the various ORFs between the different isolates of the same species as the proportion of ORFs identified in the same position across genomes of the same species (see Methods). Histograms of mean conservation for the isolates are shown in Figure 1A (for PSTVd) and Supplementary Figure S1 (for the other viroids), with a conservation score of 1 corresponding to 100% sequence identity between isolates. For most of the viroids, including PSTVd, the score is close to 1, indicating that the ORFs identified in isolates of PSTVd are highly conserved. However, this feature is not shared by all species, since some viroids, such as IRVd and CBCVd2, lack ORF sequence conservation. PVd was not included in the analysis since we only accessed one isolate of this specific viroid. The second approach was to assess the possibility of ORF existence in artificially scrambled viral genome sequences. The results are presented in Figure 1B and Supplementary Figure S2, as scatterplots of numbers of observed ORFs in real vs. scrambled genome sequences. The presence of dots above the red diagonal line of the graph corresponds to a higher tendency for the ORFs in the real sequences, whereas the presence of dots below the red line corresponds to a higher frequency for ORFs in the scrambled genome sequence. For the tested viroid species, some of them present more ORFs in their real sequence compared to the scrambled sequences (e.g., PSTVd AGVd, and HLVd), suggesting that the identified ORFs are somewhat constrained by the genomic sequence structure. Again, this is not a general feature since viroids such as CEVd, CLVd and GYSVd show more ORFs in the scrambled genome, suggesting that not all viroids have the same tendency in terms of predicted ORFs, and that even though they are in the same family, viroids may work in a different way to produce infection (Figure S2).

We also explored the possibility of ORF “hotspots”, or positions in the genome with an increased likelihood to give rise to ORFs. By projecting each identified ORF coordinate on its genome of origin, we created aggregate plots of “ORF-density” over the length of the genome for each species. We then compared the density plot with the one obtained from scrambled genomes. Results are presented in Figure 1C and Supplementary Figure S3. In PSTVd isolates, a hotspot is observed between nucleotides at positions 45 to 62, which is clearly not observed when the genome was shuffled, suggesting that this region could be important for the production of peptides. Hotspots were also observed in all viroids; however, the number as well as their distribution varies depending on the viroid species (Figure S3).

Last, we performed a structural analysis of the viroid sequences with regard to the presence of these ORFs. If a ribosome is to be attached on the viroid sequence, this is more probable to happen in a loop region than in a self-complementary base-paired sequence. For this, we calculated the presence of ORF in loops, bulges and hairpins, using published structures of viroids [18,19,55,56,57,58,59]. Although not all viroids have a solved secondary structure, most of the tested viroids have starting codons in loops, suggesting that a ribosome could attach to this region to initiate translation (Table S3).

Taken together, the above results indicate that there are ORFs present in all tested viroids, even though very few are associated with a favorable Kozak sequence. Nevertheless, there are converging indications of spatial, sequence and structural constraints associated with the identified potential ORFs. A significant percentage of these are conserved between isolates and are preferably positioned in loops, which is suggestive of an increased likelihood for translation.

To investigate this hypothesis, we focused on only one viroid, PSTVd, an important quarantine viroid, and particularly on two strains that have been widely used in different works in recent years, PSTVd^RG1^ and PSTVd^Nb^, which both contain a number of putative ORFs based on the analysis described.

### 3.2. Analysis of Potential Quasi-Species during Infections to Identify Possible Additional ORFs

**Figure 2:**
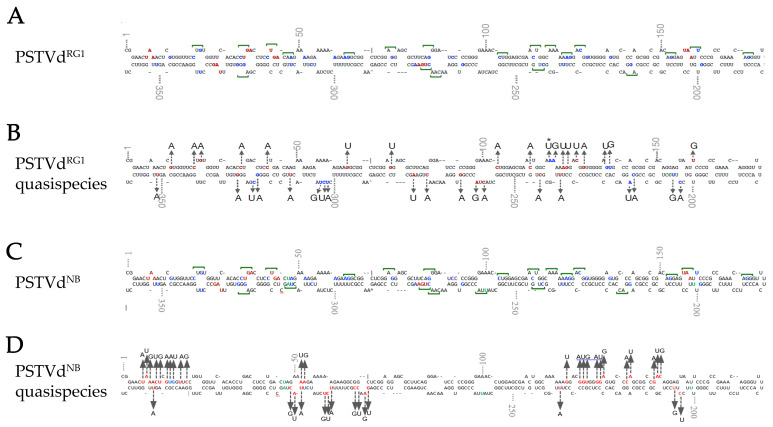
Identification of possible quasi-species using viroid-derived siRNA and total RNA NGS analysis. (**A**,**C**) To locate the potential translation start codons on the PSTVd^RG1^ and PSTVd^NB^ molecule, the in silico *detected* alternate start codons (indicated by green line over the nucleotides), the point mutation that could lead into a start codon (blue font), and the stop codons (red font) are shown on secondary structure of PSTVd. The green letters indicate the different nucleotides between PSTVd^RG1^ and PSTVd^NB^. (**B**) Analysis of sRNA derived from PSTVd^RG1^-inoculated plants revealed the presence of translation start codon (AUG) on PSTVd^RG1^ sequence. Location and changes in sequence variation that lead into the formation of potential start codons are shown on the secondary structure of PSTVd^RG1^. The red font indicates the nucleotide that was changed during infection. The two or three mutations that led into the formation of AUG are shown by blue font and an asterisk (*) indicates the nucleotide that showed both point mutation and double mutation. (**D**) Colors represent the same as in B but for PSTVd^NB^. However, only the mutations with the higher percentage range per position are represented in this figure (described in Table S4).

As already mentioned, in this analysis we used two different PSTVd strains, PSTVd^RG1^ and PSTVd^NB^, both capable of creating quasi-species during infection. A previous study showed that PSTVd may exhibit a 1/3800 to 1/7000 mutation rate [60]. A point mutation could potentially generate start codons in several regions of the PSTVd^RG1^ sequence. The PSTVd-sRNA sequences of PSTVd^RG1^-infected tomato plants (GEO Acc. No. GSM1717894), which were previously generated by Adkar-Purushothama et al. [39], were analyzed for the presence of potential start codons. The results showed a total of 143 AUG out of the 4594 PSTVd-sRNA sequences analyzed (3.1%). All the mutations that led to the formation of an AUG initiation codon are shown in Figure 2A,B.

We then performed HTS analysis using either non-infected or PSTVd^NB^-infected *N. benthamiana* plants. PSTVd^NB^ infection was confirmed by Northern blotting prior to sequencing (data not shown). HTS reads that mapped to PSTVd^NB^ were used for the identification of quasi-species. This analysis allowed the identification of a mutation likelihood expressed as percentage to be determined for each nucleotide at all genome positions (Table S4). The overall likelihood for each position in the PSTVd genome was found to be <1%; however, at positions 40 to 60 of the PSTVd genomic sequence, the mutation percentage was as high as 7% (Table S4 and Figure S4). Subsequent analysis of the mutations identified 111 putative AUG codons generated at positions where nucleotide changes were observed. Mutations with the highest probability in each position are presented Figure 2C,D. These results suggest that even if native PSTVd sequences do not possess a large number of AUG initiation codons, there is a tendency for the generation of mutations during infection/replication, which may lead to the formation of ORFs, therefore allowing the translation of peptides from viroid RNAs during the infection process.

### 3.3. The Circular Form of PSTVd Is Associated with Ribosomes

**Figure 3:**
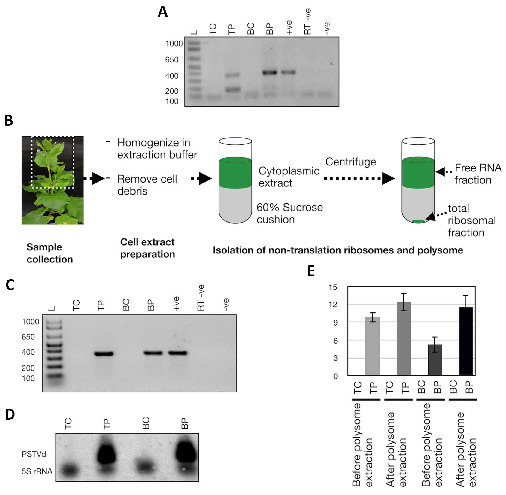
Detection of ribosome-associated PSTVd in host plants. Both Tomato cv. Rutgers and *N. benthamiana* plants were inoculated with PSTVd^RG1^. (**A**) Total RNA extracted and RT-PCR assay from these plants at 3 wpi was used to monitor the PSTVd infection. Lane L (Ladder); TC (tomato control), mock inoculated tomato plants; TP, PSTVd^RG1^ inoculated tomato plants; BC (*N. benthamiana* control), mock inoculated *N. benthamiana* plants; BP, PSTVd^RG1^-inoculated *N. benthamiana* plants; ^+^ve, RT-PCR positive control; RT ^−^ve, RT negative control and, ^−^ve, PCR negative control. (**B**) Flow chart illustrating the details of the isolation of total ribosomes from leaf samples (see Materials and Methods). The resulting precipitates were subjected to RNA purification and analyzed by (**C**) RT-PCR and (**D**) Northern blot assays. The lanes were loaded as in (**C**). (**E**) RT-qPCR to evaluate the enrichment of PSTVd^RG1^ in the ribosomes. The expression change is presented on a log2 scale. Error bars indicate the standard deviation (SD).

It has been shown before that PSTVd is found in ribosomes, but only in tomatoes [27]. In order to understand the association of PSTVd with the host ribosome during infection, tomato and *N. benthamiana* plants infected with PSTVd^RG1^ were used. PSTVd^RG1^ is known to induce severe symptoms in tomato cv. Rutgers, while *N. benthamiana* is a symptomless host [39,61]. Viroid accumulation in both tomato and *N. benthamiana* plants was confirmed by RT-PCR from the upper leaves. Both tomato and *N. benthamiana* plants showed PSTVd-specific amplicons of approximately 360 nt (i.e., the full length; Figure 3A), which was confirmed by sequencing.

Then, we investigated the presence of the viroid in ribosomes. Lysate from collected tissue was subjected to centrifugations, including ultracentrifugation on a 60% sucrose cushion (Figure 3B). RT-PCR and Northern blot analysis confirmed the presence of PSTVd in the total ribosome fraction of the infected tomato and *N. benthamiana* plants (Figure 3C,D). Additionally, RT-qPCR assays were performed on both total RNA extracts and RNA extracts derived from the total ribosomal fraction to quantify the level of viroid enrichment in the ribosomes. Higher amounts of viroid molecules were detected in the total ribosomal fraction as compared to the total RNA extract, suggesting that PSTVd is indeed enriched in the ribosomes of both tomato and *N. benthamiana* plants (Figure 3E). These results confirmed that viroids are associated with the total ribosomal fraction of infected plants.

**Figure 4:**
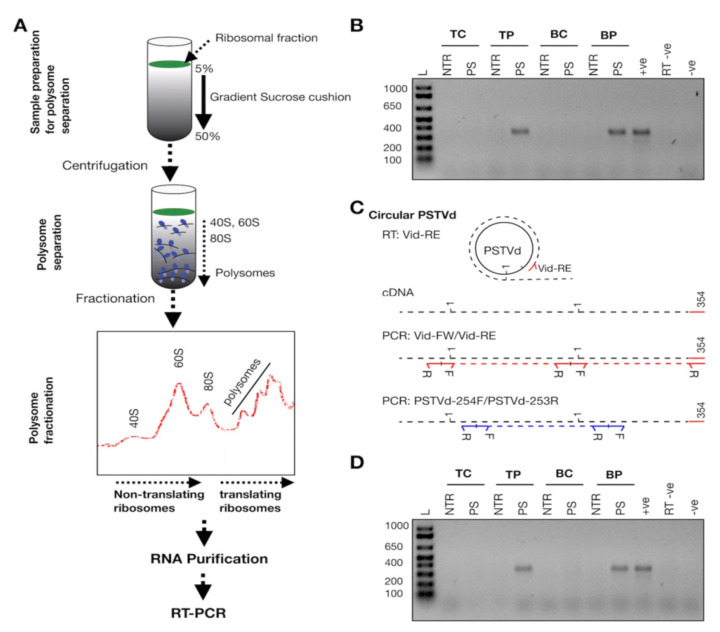
Polysome fractionation. (**A**) Flow chart illustrating the details of the separation of the 40S, the 60S and 80S ribosomes and of the polysomes. (**B**) RNA isolated from the fractionated non-translating ribosomes and from the polysomes were subjected to the RT-PCR assay using the Vid-FW/Vid-RE primer pair. Ladder (L); RNA extracted from mock inoculated tomato plants (TC), PSTVd inoculated tomato plants (TP), mock inoculated *N. benthamiana* plants (BC) and PSTVd inoculated *N. benthamiana* plants (BP). RNA extracted from non-translating ribosomes is indicated as NTR, and the RNA extracted from the polysome fraction is denoted by PS. ^+^ve, RT-PCR positive control; RT ^−^ve, RT negative control; and ^−^ve, PCR negative control. (**C**) Schematic representation of the differentiation of circular PSTVd RNA by RT-PCR assay. In the figure, the red right arrowhead indicates the Vid-FW primer, red left arrowhead indicates the Vid-RE primer, blue right arrowhead indicates the PSTVd-254F primer, and the blue left arrowhead indicates the PSTVd-253R primer. R indicates the reverse primer and F indicates the forward primer. The black dotted lines indicate the cRNA, the red dotted lines indicate the PCR product obtained with the Vid-FW/Vid-RE primer pair and the blue dotted lines indicates the PCR product obtained with the PSTVd-254F/253R primer pair. Vid-FW is complementary to nucleotide positions 355-16 of PSTVd^RG1^, Vid-RE is complementary to positions 354-336 of PSTVd^RG1^, PSTVd-254F is complementary to positions 254-273 of PSTVd^RG1^ and, PSTVd-253R is complementary to positions 253-234 of PSTVd^RG1^. The number 1 indicates the first nucleotide of PSTVd^RG1^, and the number 359 indicates the last nucleotide of PSTVd^RG1^. (**D**) PCR performed on the cDNA generated by the Vid-RE primer using the PSTVd-254F/PSTVd-253R primer set. The lanes are loaded as shown for (**B**).

However, to verify whether viroid molecules are associated with non-translating ribosomes (40S, 60S and 80S) or with polysomes, the total ribosomal fractions from leaf samples were subjected to fractionation (Figure 4A). Briefly, the isolated ribosomal fractions were dissolved in resuspension buffer and then were layered on a 5–50% sucrose gradient cushion. During centrifugation, the heavier molecules move down the sucrose gradient faster than do the lighter ones. In other words, the polysomes move towards the bottom of the tube, followed by the 80S ribosomes (monosomes), while both the 60S and 40S ribosomal subunits remain on the top of the gradient. The fractionated RNAs were grouped into non-translating ribosomes and polysomes and were subjected to RT (using the Vid-RE primer), followed by PCR amplification using the Vid-FW/Vid-RE primers. Results showed the presence of full-length PSTVd-specific amplicons were derived only from the polysome fraction of PSTVd^RG1^-inoculated tomato and *N. benthamiana* plants. No PCR amplification was detected with the RNA isolated from the non-translation ribosome fractions of the infected plants. None of the mock-inoculated plants showed any amplification (Figure 4B). The PSTVd-specific bands were cloned and sequenced in order to confirm their identity. The data presented here suggest that PSTVd is associated with polysomes in both infected tomato and *N. benthamiana* plants. It is worthy to highlight that, as described in Cottilli et al., a peak corresponding to 40S fraction is very low, suggesting that PSTVd could be affecting the 18S rRNA maturation, and therefore the 40S formation, also in *N. benthamiana* [27].

The simplest and most powerful tool with which to verify whether these polysome-associated PSTVd molecules are linear or circular RNA is cDNA synthesis using a target-specific primer followed by two independent PCRs, as described in Figure 4C. If the target is circRNA, both PCRs should yield amplicons equivalent to the full-length targets, whereas if the target is monomeric linear, only one PCR will yield a full-length target. Hence, second full-length PCR amplification was performed using a new set of primers (PSTVd-254F/PSTVd-253R) on the RT product which was synthesized using the Vid-RE primer. Results revealed the presence of full-length PSTVd amplicons in the polysome fraction of PSTVd inoculated plants (also verified by sequencing), but not in either the ribosome fraction or in the mock-inoculated plants (Figure 4D). Taken together, these results suggest that circular PSTVd molecules are found in translating ribosomes of both tomato and *N. benthamiana* plants.

### 3.4. In Vitro Translation of PSTVd

**Figure 5:**
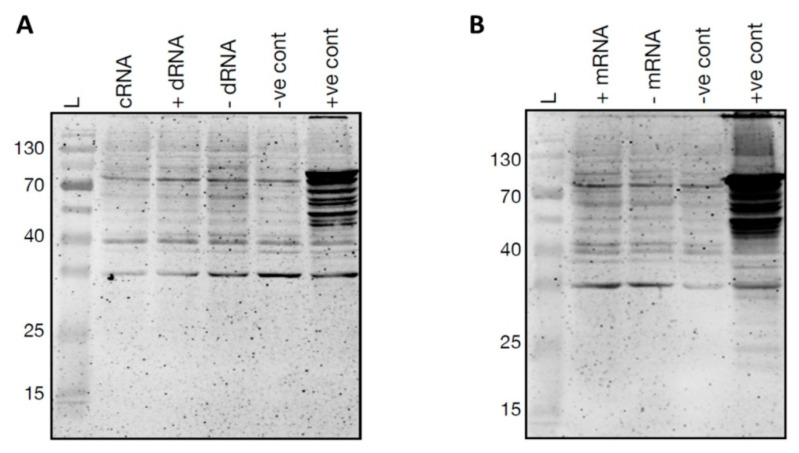
In vitro translation of PSTVd^RG1^. In vitro translation of (**A**) circular RNA (cRNA), dimeric (+) PSTVd RNA (+ dRNA), dimeric (−) PSTVd RNA (-dRNA) and (**B**) monomeric (+) PSTVd RNA (+ mRNA), monomeric (−) PSTVd RNA (- mRNA). A reaction mixture without any template RNA was used as negative control (^−^ve cont), and luciferase control RNA was used as the positive control (^+^ve cont).

In order to verify potential start codons, in vitro translation assays were performed using the wheat germ extract system with the idea of verifying whether or not PSTVd^RG1^ ORFs could be translated into peptides. For this purpose, in vitro-generated circular (+) PSTVd and both (+) and (−) monomeric and dimeric PSTVds RNAs were prepared using a synthetic PSTVd^RG1^ sequence. The positive control (i.e., luciferase control RNA) produced a high intensity band, while none of the tested viroid transcripts permitted the detection of peptides by immunoblot assays (Figure 5). These experiments were repeated under many different conditions, including the use of various concentrations of both magnesium (2 to 5 mM MgCl_2_) and potassium (50 to 150 mM KCl), as well as of various incubation times (60–120 min) and temperatures (25 °C to 30 °C). Regardless of the conditions tested, it was not possible to detect the synthesis of any peptides derived from the PSTVd template.

### 3.5. Using Mass Spectrometry to Identify PSTVd Produced Small Peptides

**Figure 6:**
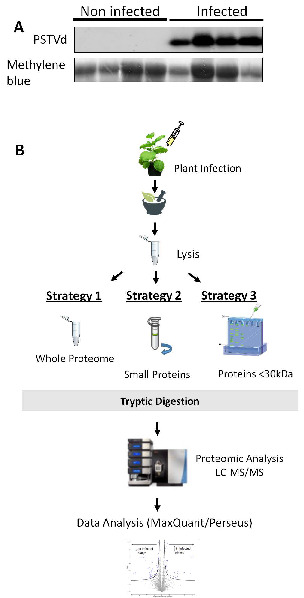
Experimental design for MS experiments. (**A**) Northern blot for the detection of PSTVd^NB^ in *N. benthamiana* plants. Total RNA staining (methylene blue) was used as loading control. (**B**) Three different strategies were followed in this study. In strategy 1, total lysate from both infected and non-infected plants was used for further MS analysis. In strategy 2, total lysate was filtered through specialized column to keep only small peptides, and then proceed with MS analysis. In strategy 3, a 15% polyacrylamide gel was used to separate proteins and only proteins smaller than 30 kDa were kept for further MS analysis.

**Figure 7:**
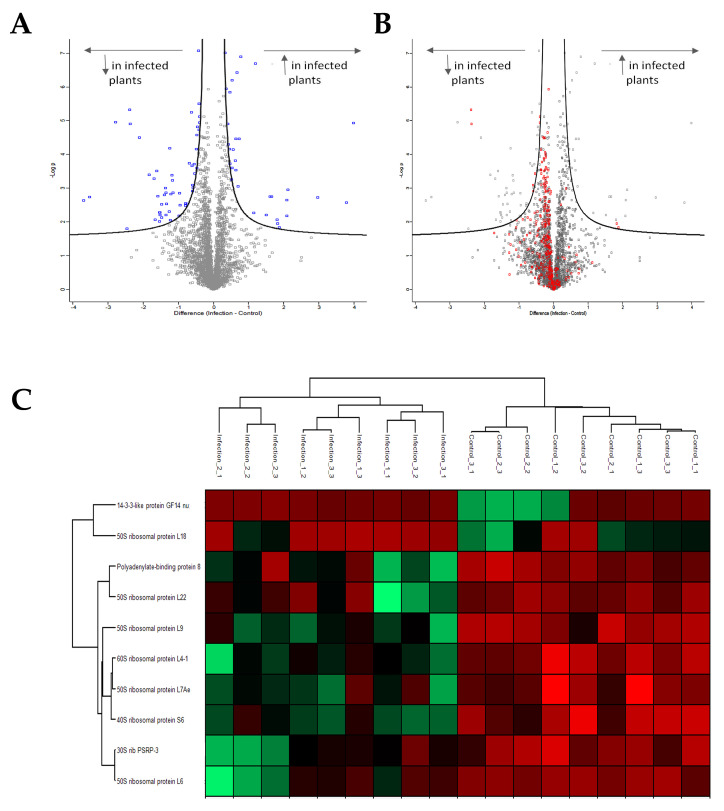
MS analysis. (**A**) Volcano plot showing all proteins affected in PSTVd-infected *N. benthamiana* plants. In total, 85 proteins were shown to be statistically affected. (**B**) Volcano plot showing proteins related to the translation mechanism affected upon PSTVd infection of *N. benthamiana* plants. (**C**) Heat map of proteins related to translation statistically affected by the infection of PSTVd. All graphs were created using the Perseus 1.6.10.43 software [52]. More details in Table S5.

To study in vivo possible PSTVd peptide production, we performed MS analysis in infected plants. *N. benthamiana* plants were inoculated with PSTVd^NB^ and 4 wpi leaves were collected and tested for viroid presence (Figure 6A). Since we have used PSTVd^NB^, the expected peptides to be produced were known and are shown in Table 3. We selected and performed three biological and three technical replicates for not infected and PSTVd-infected plants. We identified 3730 different proteins, and after filtering (see Materials and Methods), we kept 3227 proteins for further analysis, presented in Table S5. We first focused on the analysis of the proteins found in order to validate the MS technique. After statistical analysis, 85 proteins were identified as having their expression altered by PSTVd infection and are shown in a volcano plot (Figure 7A) as well as in detail in Table 4. The log2 difference is derived from the statistical comparison of the LFQ intensities between the two groups (infected samples vs. control samples). In order to verify the results, we looked at older published data [28]. Proteins such as oxygen-evolving enhancer protein 2 (OEE2) or pathogen-related protein 10 (PR10) were found in our experimental set as statistically significantly altered by PSTVd, as has been previously described for CEVd [28]. Therefore, we considered that our results were of good quality to be used for further analysis.

By analyzing the ontology of the identified proteins in detail (Table 4), it was revealed that certain proteins involved in metabolism and stress are influenced by the viroid infection. In addition, most of the affected proteins are located in the cytoplasm or in the chloroplast and not in the nucleus (Figure S5). Furthermore, an important number of proteins involved in translation seem to be affected. In Figure 7B, the red dots of the Volcano plot represent all identified proteins involved in translation, and it is obvious that there is a tendency for under-expression of these proteins upon viroid infection. Some of these proteins have been found with statistically significant changes (Figure 7C). This result suggests that translation is leading to a shut down during viroid infection.

Then, we focused on the presence of small PSTVd peptides. Even though in the performed analysis we were able to recognize a significant number of small peptides (as small as 3kDa), none of the in silico-predicted PSTVd microproteins were positively identified. Therefore, we proceeded with two alternative strategies (Figure 6B). We reasoned that the previous highly complex in-protein experiment may have masked some small peptides due to the large number of cellular proteins found in the lysate. For that reason, we opted for the filtration of the lysate to enrich our samples for low-molecular-weight proteins (Figure 6B). We also performed a third strategy, where we performed a 15% SDS-PAGE gel, cut bands under 30kDa and repeated the proteomic analysis. The data analysis of the above strategies was performed using both specific trypsin digestion as well as the non-stringent “unspecific digestion” alternative. Unfortunately, even though small peptides were identified originating from other proteins, we were not able to identify any of the predicted PSTVd peptides.

## 4. Discussion

Since the discovery of viroids, it has been generally accepted that they do not encode ORFs. However, recent research developments suggest that viroids can bind to ribosomes [27]. In addition, endogenous circRNAs have the capacity of being translated and produce small peptides called micropeptides [8,9,10,11]. With this in mind, we decided to revisit the idea that viroid RNAs are not translated by using distinct and sensitive techniques.

We performed thorough bioinformatics analysis using 30 different *Pospiviroidae* species, including 2441 published isolates. We showed that all tested viroid sequences contain small ORFs with mean sizes of putative polypeptides ranging between 3 and 15 kDa. We have considered ORFs that started with AUG or non-AUG starting codons [2]; however, very few of them presented a favorable Kozak sequence that would predict enhanced translation. The presence of these ORFs does not appear to be random since, as suggested by the spatial preference of ORFs to occur in the same position across genomes. Finally, by analyzing all viroid isolates, we determined ‘hotspots’ usually found in structurally loose regions of the genome. For PSTVd the hotspot was mostly located between positions 40 to 60 in the pathogenicity region. These analyses provide evidence that viroid genomes possess potential ORFs that could be translated.

The production of microproteins from small ORFs, including from circRNA, has been described previously [8,9,10,11]. Precursors of miRNAs, which have been previously proposed to have similar structural features to viroids, have also been found to interact with ribosomes and produce micropeptides ranging from 4 to 60 aa [62,63,64]. ORF translation from UTR has also been produced by uORFs (upstream ORFs in the 3′UTR) or sORFs (small ORFs generally in 5′UTRs). Most uORFs are found upstream of major mRNA ORFs and are most often initiated using an AUG start codon. However, almost 50% of uORFs have been found to start from non-AUG start codons [65]. The production of peptides from uORFs has been found essential in translation since it can either enhance translation (e.g., ribosomal shunt) or reduce it [66,67]. Finally, circular RNA satellites, which are small pathogens sharing a few common characteristics with viroids, have been found capable of producing small peptides [21].

In this work, we have specifically focused on PSTVd to study the possible production of peptides by viroids in the two different strains used in this work, PSTVd^RG1^ and PSTVd^NB^. Although there was no AUG present, there were a few non-AUG starting codons, allowing the production of peptides ranging from 3 to 204 aa for PSTVd^NB^ and from 2 to 61aa for PSTVd^RG1^. However, upon infection, a significant number of point mutations are produced (3% and 7% depending on the system) as has been shown before [60], also generating AUG starting codons, that can be used for initiation of translation. However, the number of recognized quasi-species with these mutations is relatively small to significantly affect viroid biology.

It has been shown that CEVd genomic RNA as well as viroid-derived siRNAs have been localized in ribosomes [27], suggesting that *pospiviroidae* species have the tendency of accumulating in ribosomes. In this work, we have shown that the circular PSTVd genome localizes in ribosomes in *N. benthamiana* and tomato plants too. Therefore, applying a combination of new and older techniques, we aimed to test the hypothesis that viroids can be translated. We first performed in vitro experiments, but no translation products were found in any of the different conditions tested. Older experiments using both PSTVd and CEVd in in vitro translation experiments showed similar results [22,23]. In addition, analogous experiments in viroid PLMVd of the *Avsunviroidae* family again did not produce any peptides (F. Cote and J.P. Perreault, unpublished results). Taken together, these results suggest that no peptides are produced in cell-free in vitro systems. Nevertheless, this system has some limitations, including low protein yield [68], and therefore we cannot exclude the possibility that peptides may be produced but not detected. Consequently, we opted for an in vivo experiment to look for peptides using a different technique.

We performed proteomic analysis in lysates of PSTVd-infected *N. benthamiana* plants, using a robust dataset containing three biological replicas and three technical replicas. We showed altered expression of 85 proteins during PSTVd infection. Some, such as OEE2 and PR10, have also been described previously, suggesting that our analysis was accurate [28]. We found that an important number of PSTVd deregulated proteins are localized in the cytoplasm. In addition, we found that apart from proteins usually affected upon infection, such as stress proteins or proteins related to different metabolic pathways, proteins related to the translation mechanism were also influenced, showing a trend of under-expression. This phenomenon could be related to ribosomal stress. It has been proposed before that during CEVd infection, ribosomal biogenesis in tomato plants was affected [27]. Downregulation of proteins related to translation could also be a result of a translation shut-off. Viruses benefit from a decrease in the translation of endogenous transcripts as this protects them from defense-related proteins. In addition, they may divert translation to their own benefit [69]. This can be achieved by different mechanisms such as influencing translation initiation factors or even cleaving endogenous mRNAs. Hence, the most common ‘strategy’ used by viruses is to either bind or affect the phosphorylation translation initiation or elongation factors [69]. It has been proposed before by independent studies that CEVd, PSTVd and PMLVd bind eIF1A [28,29]. Other factors such as eEF2 and eIF5A have been found to be influenced by CEVd infectivity [27], suggesting that viroids may decrease the translation rate in order to gain time for establishing host propagation.

From the standard LC-MS/MS lysate analysis, no PSTVd-expressed microprotein was identified. We reasoned this could be due to the large number of proteins identified, that could in a way ‘mask’ small peptides. Therefore, we have opted firstly for a filtering of the lysate, keeping only small peptides, and, secondly assessed proteins smaller than 30 kDa following electrophoresis, using LC-MS/MS. Again, both strategies failed to identify PSTVd-derived peptides. It cannot be excluded that technical limitations may be responsible for this. One possibility is that these peptides are extremely hydrophilic, making them difficult to be detected by the LC-MS/MS technique. Then again, we have tested the predicted peptides with a specific software for hydrophobicity, and they were found adequate for LC-MS/MS (data not shown). Another issue could be the low quantity of the produced peptides. Yet, as shown in a Northern blot, the quantity of viroid present at 4 wpi is high enough to assume that if a peptide is produced by each molecule, then its quantity should be detectable. Another possibility could be a fast peptide degradation procedure that would increase the difficulty to obtain a peptide fragment in LC-MS/MS, even though a protease inhibitor was added into the lysis buffer. We cannot also exclude that a probable PSTVd peptide could be retained in a specific cellular domain that we cannot obtain using this work specific conditions. Finally, the used lysis buffer could be improved for small peptides as it was recently published [70].

## 5. Conclusions

Our results suggest that even though viroids are present in ribosomes and have ORFs which are potentially translatable, no peptide was identified using either in vitro or in vivo translation experiments. Therefore, viroids may be ‘using’ ribosomes for reasons other than translation. One possibility could be binding to ribosomes for protection. It has been shown before that the ribosome protects the portion of RNA enclosed within its subunits [71,72]. Although usually only around 35 nt are protected, more than one ribosome can typically be found associated with an mRNA [72]. Therefore, we could speculate that through binding to PSTVd RNAs, multiple ribosomes can provide protection from the action of different cellular nucleases. An alternative explanation may be related to the movement of viroid RNAs. Ribosomes localize at the surface of the endoplasmic reticulum, the mitochondria, as well as freely in the cytosol. Cytosolic ribosomes are suggested to use microtubules to circulate in cells [73], so it could be speculated that viroids use ribosomes to move within the cell. Finally, another possibility could be that viroids are binding to ribosomes to hijack the translation mechanism. However, if and to what extent the interaction of viroid ribosomes is related to viroid pathogenicity remains unclear. Taken together, this study shows that even though ORFs are present in viroids, in our experimental conditions, they do not seem to be translated. Nevertheless, viroids may utilize ribosomes for a different reason. Further experimentation is needed to test such a hypothesis.

## Figures and Tables

**Table 1:** Identified Small Peptides in Viroids.

Viroids	Abbreviations	Number of Different Peptides	Mean of Peptides Molecular Weight (Da)	Deviation of Peptides Molecular Weight (Da)	Mean Number of Nucleotides	Deviation of Number of Nucleotides
Apple Dimple viroid	ADFVd	112	4,985,472	3,839,693	124,488	92,301
Apple Scar Skin viroid	ASSVd	403	5,357,067	3,545,363	13,651	87,982
Australian Grapevine viroid	AGVd	385	5,797,681	3,301,071	146,785	82,668
Chrysanthemum Stunt viroid	CSVd	391	5,696,392	3,064,454	138,372	71,622
Citrus Bark Cracking viroid	CBCVd	109	5,614,057	3,736,423	139,106	89,363
Citrus Bent leaf viroid	CBLVd	197	4,559,265	3,222,127	118,054	8299
Citrus Dwarfing viroid	CDVd	317	4,235,954	2,457,568	1,043,122	57,357
Citrus Exocortis viroid	CEVd	717	8,908,622	414,267	22,654	103,926
Citrus viroid V	CVd-V	79	4,790,307	3,350,296	123	83,618
Citrus viroid VI	CVd-VI	80	4,946,414	3,800,323	122,963	92,295
Coconut cadang-cadang viroid	CCCVd	27	848,874	4,918,339	209,777	116,172
Columnea Latent viroid	CBVd1	290	7,315,868	5,184,612	187,693	134,273
Coleus Blumei viroid 1	CBVd2	26	5,725,483	295,522	144,387	71,306
Coleus Blumei viroid 2	CBVd3	14	14,849,266	4,436,371	376	133,152
Coleus Blumei viroid 3	CBVd5	12	5,500,916	4,175,932	13,475	101,042
Coleus Blumei viroid 5	CBVd6	12	76,025	3,621,197	196,285	93,803
Coleus Blumei viroid 6	CLVd	7	11,385,714	8,186,838	287,571	208,934
Dahlia Latent viroid	DLVd	10	3024	2,759,504	78	71,233
Grapevine Yellow Speckle viroid 1	GYSVd1	404	70,593	381,389	177,653	94,235
Grapevine Yellow Speckle viroid 2	GYSVd2	202	9564	501,981	236,137	121,745
Hop Latent viroid	HLVd	48	6,968,053	4,348,142	175,553	110,481
Hop stunt viroid	HSVd	1187	797,481	5,295,964	19,715	129,347
Iresine viroid 1	IrVd	36	8,082,925	4,206,529	20,985	10,268
Mexican Papita viroid	MPVd	40	9,292,166	4,819,751	229,214	116,252
Pear Blister Canker viroid	PBCVd	199	442,312	303,035	11,375	76,997
Pepper Chat Fruit viroid	PCFVd	133	85,162,875	443,507	2135	108,787
Persimmon viroid 2	PVd	8	405,225	2,535,428	1035	6605
Potato Spindle Tuber viroid	PSTVd	612	10,691,688	6,520,371	269,441	16,247
Tomato Apical Stunt viroid	TASVd	102	684,758	408,641	16,863	1001
Tomato Chlorotic Dwarf viroid	TCDVd	56	5943	470,731	14,877	117,745

**Table 2:** Presence of Kozak Frame in Viroid Species.

Viroid.	Number of Isolates
PSTVd	17
CEVd	1
CSVd	8
CLVd	1

**Table 3:** Predicted Peptides for PSTVd^NB^.

Start Site	Stop Site	Amino Sequence	Length	Molecular Weight
33	42	LTSSTOP	3	355
51	156	KKKEGGSEERFRDPRGNLERTGKKGRWGVPSGRQESTOP	35	4621
58	311	KKAARRSASGIPGETWSELAKKDGGECPAADRSNSRRNRVFTLPFFGFPSSRPQDHPSPPLRCRFGYYPVETTEAPENRFFSILLAPGRGCLALGTAVGSSELNSWFLWFTPDLLTRKEKRRRLGGALQGSPGKPGANWQKRTVGSAQRPTGVIPAETGFSPFLSSGFLPRARRTTPRPLCAVASAITRWKQLKLPRTAFSLSYSTOP	204	24,375
243	276	LSLRLLPGGNNSTOP	11	1332
324	34	RVFSPWNRSWFLGTKLVVPVVHTSTOP	23	3119
338	6	LEPQLVPRNSTOP	9	1208

**Table 4:** List of Identified Proteins Affected During Viroid Infection.

C: Human-Readable-Description		
Sol Genomics	Benth Genome	Log2 Difference (Infected Samples-Control Samples)
**Signaling**		
Protein phosphatase 2C family protein	Probable protein phosphatase 2C 55	−1.3518609
Peroxiredoxin-2B	Peroxiredoxin-2B (probable),thioredoxin peroxidase 1	0.4045086
SIT4 ph isoform 1 [Theobroma cacao];SIT4 phosphatase-associated family protein SIT4 phosphatase-associated family protein	serine threonine-protein phosphatase 6 regulatory subunit 3-like isoform x1	−0.7918434
Pleckstrin homology (PH) domain-containing protein/lipid-binding START domain-containing protein	protein enhanced disease resistance 2-like isoform x1	−1.538706
Ankyrin repeat domain-containing protein 6	Protein LHCP TRANSLOCATION DEFECT (probable)	−1.6666896
Protein kinase superfamily protein	serine threonine-protein kinase at5g01020	−2.4678752
**Methylation**		
S-adenosyl-L-methionine-dependent methyltransferases superfamily protein	Probable methyltransferase PMT26 (probable)	−0.5842584
sterol methyltransferase 2	24-methylenesterol c-methyltransferase 2-like	−1.4808227
Sterol 24-C-methyltransferase;sterol methyltransferase 1	cycloartenol-c-24-methyltransferase 1-like	−1.2608874
**Translation**		
Polyadenylate-binding protein;Polyadenylate-binding protein 1;Polyadenylate-binding protein 5;Polyadenylate-binding protein 8	Polyadenylate-binding protein RBP45B (probable)	−2.3749559
50S ribosomal protein L6	60S ribosomal protein L9-1 (probable),60s ribosomal protein l9-1-like	−0.5959295
50S ribosomal protein L18	50S ribosomal protein L18, chloroplastic (probable)	1.8289892
40S ribosomal protein S6	40S ribosomal protein S6 (probable)	−0.3994783
30S rib PSRP-3 [Prochlorococcus marinus str. SB];rib PSRP-3/Ycf65 [Halothece sp. PCC 7418]	30s ribosomal protein chloroplastic-like	−1.1850082
Polyadenylate-binding protein 8	33 kDa ribonucleoprotein, chloroplastic (probable)	−0.5907972
50S ribosomal protein L7Ae	h aca ribonucleoprotein complex subunit 2-like protein	−0.5377388
50S ribosomal protein L9	50S ribosomal protein L9, chloroplastic (probable)	−2.379722
60S ribosomal protein L4-1	60s ribosomal protein l4-1-like	−0.3906072
Nucleolar protein 58	probable nucleolar protein 5-2	−0.6824243
**Metabolism**		
Glucose-1-phosphate adenylyltransferase family protein	Glucose-1-phosphate adenylyltransferase large subunit 1 (probable)	2.093622
N-succinylglutamate 5-semialdehyde dehydrogenase	delta-1-pyrroline-5-carboxylate dehydrogenase mitochondrial	0.5348445
Threonine synthase	Threonine synthase, chloroplastic (probable)	−0.9644074
2-dehydro-3-deoxyphosphoheptonate aldolase (3-deoxy-d-arabino-heptulosonate 7-phosphate synthase) [Medicago truncatula] gb|AES98110.1| phospho-2-dehydro-3-deoxyheptonate aldolase [Medicago truncatula]	Phospho-2-dehydro-3-deoxyheptonate aldolase 2, chloroplastic (probable)	−0.5528278
2-dehydro-3-deoxyphosphoheptonate aldolase (3-deoxy-d-arabino-heptulosonate 7-phosphate synthase) [Medicago truncatula] gb|AES98110.1| phospho-2-dehydro-3-deoxyheptonate aldolase [Medicago truncatula]		−1.2349175
alanine aminotransferase 2	alanine aminotransferase 2	−1.469876
Cytochrome P450 superfamily protein;Cytochrome P450 superfamily protein	sterol 14-demethylase-like, sterol 14-demethylase-like	−0.9758828
3-isopropylmalate dehydratase large subunit	aconitate cytoplasmic	−0.6276336
Isoflavone reductase homolog	isoflavone reductase homolog	0.5225232
S-adenosylmethionine synthase 3	s-adenosylmethionine synthase 2-like	−0.4216976
alanine aminotransferase 2	Glutamate--glyoxylate aminotransferase 2	0.3580829
Aspartate aminotransferase, mitochondrial;aspartate aminotransferase 5	Aspartate aminotransferase, chloroplastic (probable)	0.3257779
ATP-dependent zinc metalloprotease FtsH	cell division cycle protein 48 homolog	−1.3560431
adenylate cyclase [Zea mays]	triphosphate tunel metalloenzyme 3 isoform x1	−0.9491795
Acetolactate synthase	acetolactate synthase chloroplastic-like	−0.4911346
Methylenetetrahydrofolate reductase 1	Probable methylenetetrahydrofolate reductase (probable)	−0.4068656
ornithine carbamoyltransferase	Pistil-specific extensin-like protein (probable)	−1.4021558
Linoleate 9S-lipoxygenase 6	lipoxygenase	−1.3354193
GDSL esterase/lipase	gdsl esterase lipase at1g29670-like	−1.5272558
GDSL esterase/lipase	gdsl esterase lipase at5g33370-like	0.6638832
Gamma-glutamyl phosphate reductase	delta-1-pyrroline-5-carboxylate synthase-like isoform x2	0.5598941
senescence-associated protein [Arabidopsis thaliana];Thiosulfate sulfurtransferase GlpE	Rhodanese-like domain-containing protein 15, chloroplastic (probable)	1.1901923
3-phosphoshikimate 1-carboxyvinyltransferase	3-phosphoshikimate 1-carboxyvinyltransferase 2	−1.5891065
aminoacyl peptidase [Xanthomonas axonopodis]	probable glutamyl chloroplastic isoform x1	1.1461201
Eukaryotic aspartyl protease family protein	aspartic proteinase nepenthesin-1-like	−2.7812869
Ribulose-1,5 bisphosphate carboxylase/oxygenase large subunit N-methyltransferase, chloroplast precursor, putative [Ricinus communis] gb|EEF30179.1| Ribulose-1,5 bisphosphate carboxylase/oxygenase large subunit N-methyltransferase, chloroplast precursor, putative [Ricinus communis]	ribulose- bisphosphate carboxylase oxygenase large subunit n- chloroplastic	1.5090357
iron-binding protein [Pyrus pyrifolia]	Ferritin-1, chloroplastic (probable)	−1.68272
iron-binding protein [Pyrus pyrifolia]	Ferritin-2, chloroplastic (probable)	−1.8307396
**Stress**		
Protein GrpE	grpe protein mitochondrial	−1.6075834
Annexin D1	annexin d1-like	0.4671304
Heavy metal transport/detoxification superfamily protein	pollen-specific leucine-rich repeat extensin-like protein 1	3.9868143
Universal stress protein A-like protein	Universal stress protein A-like protein	0.5305178
Glutathione S-transferase U8	glutathione transferase gst 23-like	0.7272462
IMP dehydrogenase/GMP reductase [Synechocystis sp. PCC 6714]	probable uncharacterized protein ycf23-like	0.6426404
Glutamate dehydrogenase B	glutamate dehydrogenase b	0.7839004
Plastid-lipid associated protein PAP / fibrillin family protein	fibrillin 1 protein	0.6953284
membrane related		
Pyrophosphate-energized vacuolar membrane proton pump	pyrophosphate-energized vacuolar membrane proton pump 1-like	3.7827212
CASP-like protein 4D1	casp-like protein 4d1	−2.1034703
Transmembrane emp24 domain-containing protein A	Transmembrane emp24 domain-containing protein p24beta3-like	−1.2165958
Aspartic proteinase;Aspartic proteinase A1	aspartic proteinase-like	−1.2461262
GRIP and coiled-coil domain-containing protein, putative [Ricinus communis] gb|EEF50040.1| GRIP and coiled-coil domain-containing protein, putative [Ricinus communis]	uncharacterized abhydrolase domain-containing protein ddb_g0269086-like	−0.4647138
Signal peptidase complex subunit 3B	Signal peptidase complex subunit 3B (probable)	−1.5427202
Aquaporin-2;Aquaporin-like superfamily protein	aquaporin tip2-1-like	−0.4083201
ATP synthase subunit a, chloroplastic	Proteasome subunit beta type-1 (probable)	1.6636887
**Photosynthesis**		
Plastocyanin A’/A’’	plastocyanin a a	−1.5127553
Oxygen-evolving enhancer protein 2-1, chloroplastic	oxygen-evolving enhancer protein 2- chloroplastic	0.4273438
Oxygen-evolving enhancer protein 2, chloroplastic	oxygen-evolving enhancer protein 2- chloroplastic	0.4711001
Photosystem II CP47 reaction center protein	Photosystem II CP47 chlorophyll apoprotein (probable), photosystem ii 47 kda protein	−3.5313221
Glutamyl-tRNA reductase-binding protein, chloroplastic;pyridoxamine 5’-phosphate oxidase [Mycobacterium abscessus]	glutamyl-trna reductase-binding chloroplastic	−0.4534192
**Protein folding**		
Thioredoxin superfamily protein [Theobroma cacao] gb|EOX91756.1| Thioredoxin superfamily protein [Theobroma cacao];Thioredoxin superfamily protein	prostamide prostaglandin f synthase	0.6359446
Calnexin homolog	calnexin homolog	−0.8004602
transcription		
potyviral VPg interacting protein 2 [Phaseolus vulgaris]	Protein OBERON 2 (probable)	2.9606433
glycine-rich RNA-binding protein 2	glycine-rich rna-binding, glycine-rich rna-binding	−1.1653803
Chromodomain-helicase-DNA-binding protein 1	ATP-dependent helicase BRM (probable)	2.1251746
**Defence proteins**		
Major pollen allergen Bet v 1-D/H	pathogenesis-related protein sth-2-like	0.6196096
MLP-like protein 31	pr-10 type pathogenesis-related protein	1.6111262
**Diverse roles or unknown roles**		
Peptidyl-prolyl cis-trans isomerase-like 1	peptidyl-prolyl cis-trans isomerase chloroplastic	0.4489725
protein of unknown function (DUF1995) [Leptolyngbya sp. PCC 6406]	probable uncharacterized protein LOC104217371	−0.6120353
14-3-3-like protein GF14 nu;14-3-3-like protein GF14-C	14-3-3-like protein a, 14-3-3 protein 4-like	1.8770383
Bifunctional inhibitor/lipid-transfer protein/seed storage 2S albumin superfamily protein	36.4 kDa proline-rich protein (probable)	−3.6962613
Fructokinase-2	fructokinase 2	−0.4746384
ubiquitin family protein	ubiquitin domain-containing protein dsk2b-like isoform x1	−1.3615259
26S proteasome non-ATPase regulatory subunit 12 homolog A	26s proteasome non-atpase regulatory subunit 12 homolog a-like	−1.1566838
DNA replication licensing factor MCM2	DNA replication licensing factor mcm2 (probable)	−0.7461614

## Data Availability

The mass spectrometry proteomics data have been deposited to the ProteomeXchange Consortium via the PRIDE [74] partner repository with the dataset identifier PXD030755.

## Supplementary Materials

The following are available online at https://www.mdpi.com/article/10.3390/cells11020265/s1, Figure S1: Conservation rate in viroid species, Figure S2: Comparison between bioinformatically shuffled genome and real genome for viroids, Figure S3: Presence of ‘hotspots’ in viroid genomes, Figure S4: Nucleotide mutation rate for PSTVd, Figure S5: GO enrichment analysis using PlantRegMap, focusing of cellular compartment, Table S1: Viroids and strains used for this analysis (by NCBI), Table S2: Primers used in this study, Table S3: ORF present in different part of viroid structure, Table S4: Presence of alternative nucleotides in different PSTVd positions, Table S5: Proteins identified by MS in PSTVd infected vs. Healthy N. benthamiana plants.
