# Supplementary figures and images for: Revisiting the Non-Coding Nature of Pospiviroids
Source: Cells. 2022 Jan 13;11(2):265. doi: 10.3390/cells11020265 (PMC8773695; doi:10.3390/cells11020265)

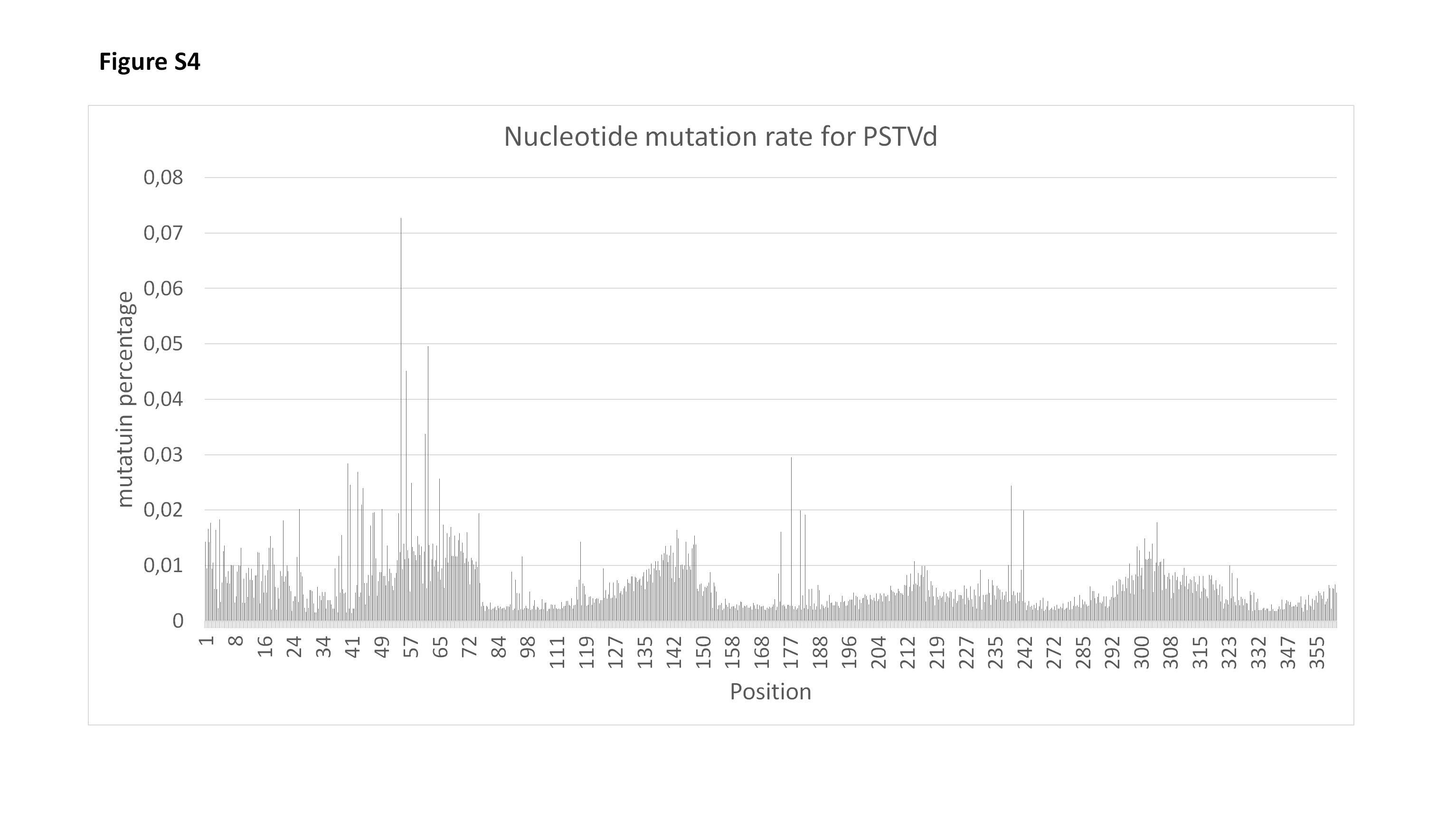

Supplement: Supplementary file 1 [file cells-11-00265-s001.zip › Figure_S4.jpg]

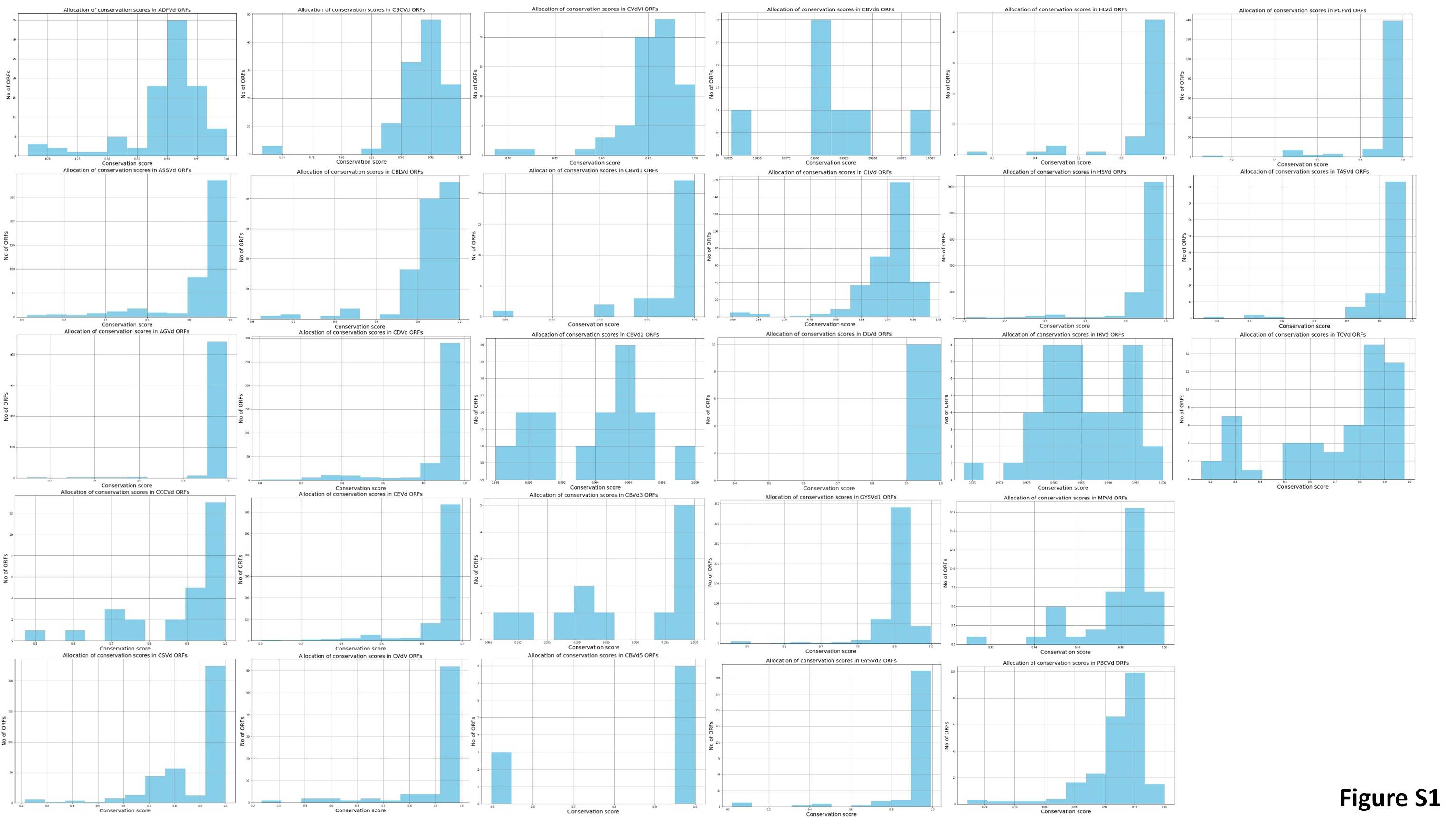

Supplement: Supplementary file 1 [file cells-11-00265-s001.zip › Figure_S1.jpg]

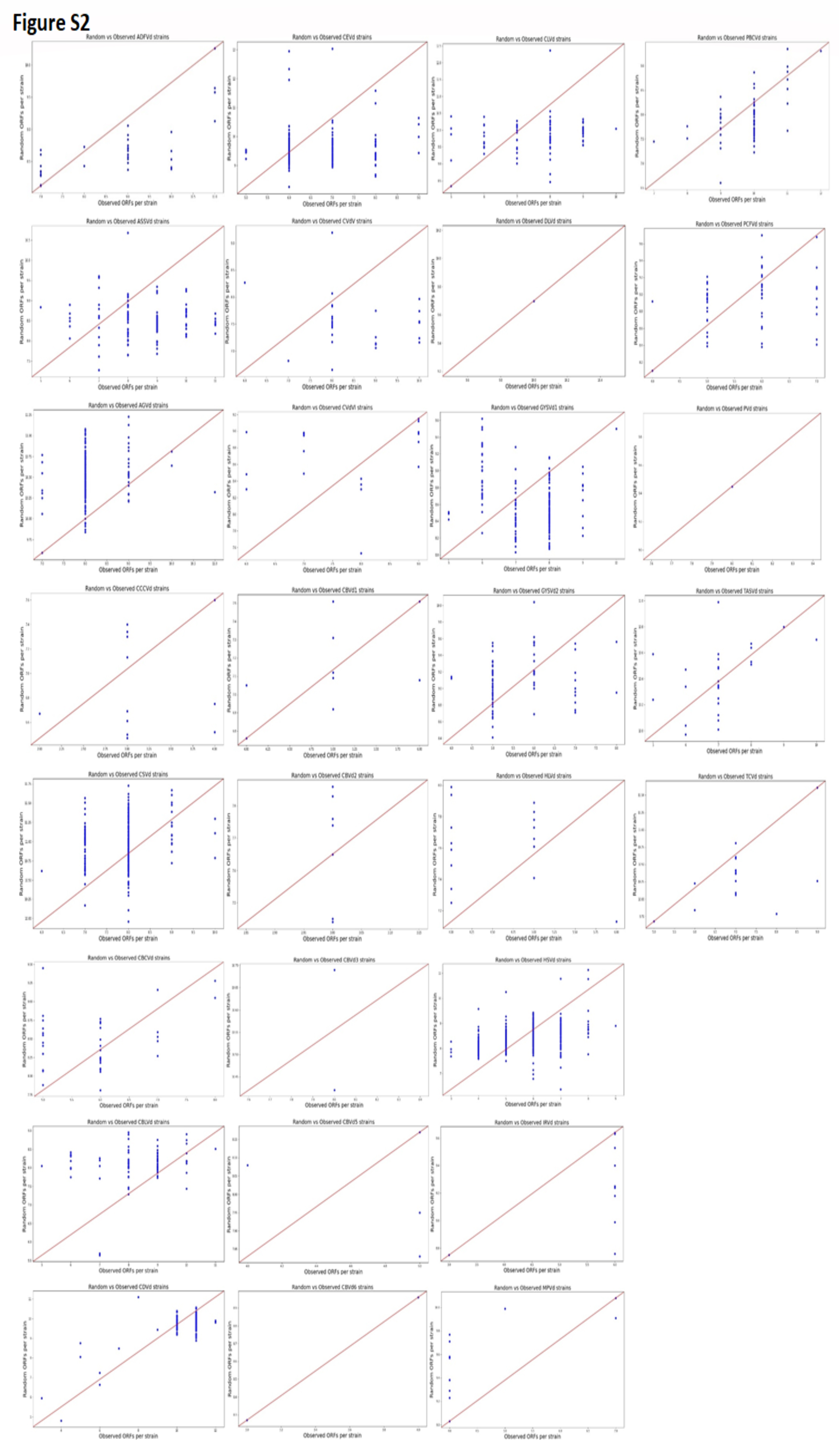

Supplement: Supplementary file 1 [file cells-11-00265-s001.zip › Figure_S2.jpg]

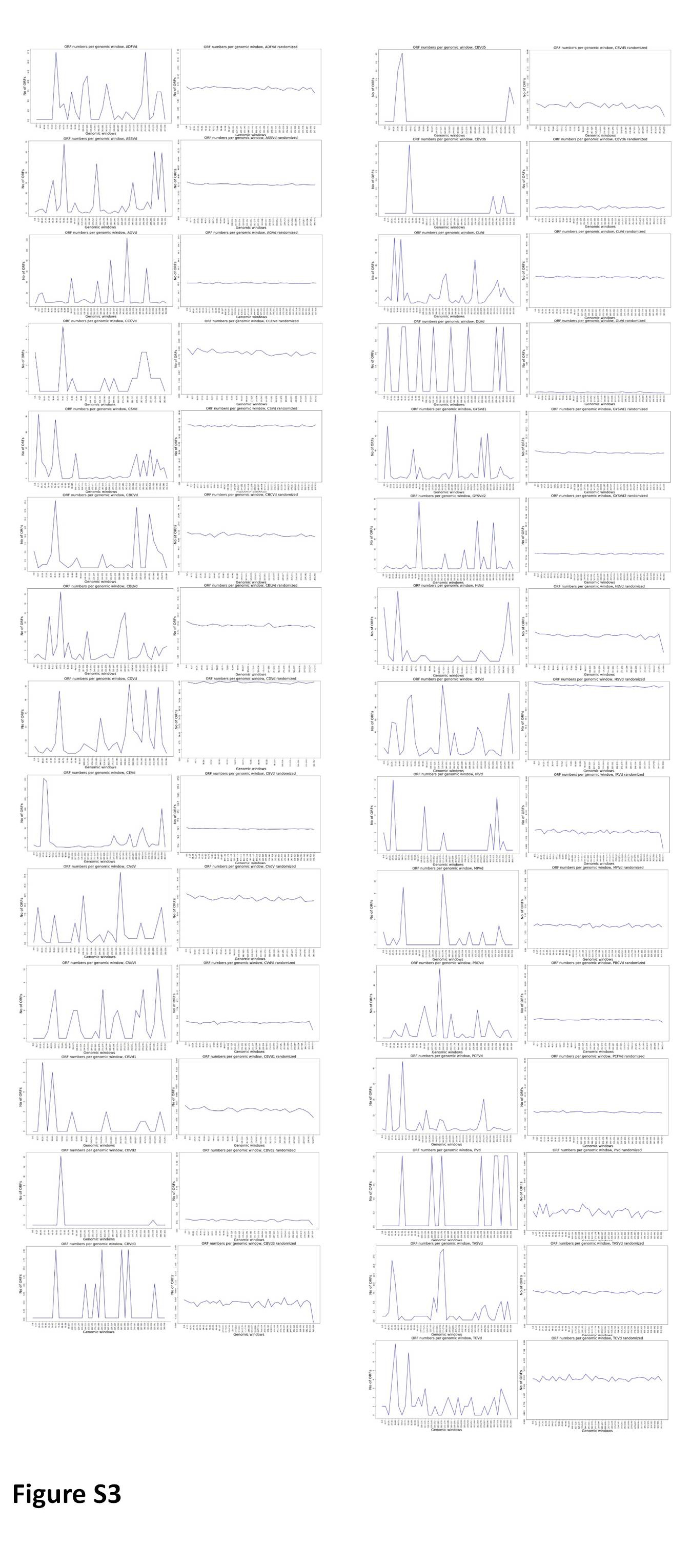

Supplement: Supplementary file 1 [file cells-11-00265-s001.zip › Figure_S3.jpg]
